# Efficacy of probiotics/synbiotics supplementation in patients with chronic kidney disease: a systematic review and meta-analysis of randomized controlled trials

**DOI:** 10.3389/fnut.2024.1434613

**Published:** 2024-08-06

**Authors:** Chang Liu, Letian Yang, Wei Wei, Ping Fu

**Affiliations:** Department of Nephrology, Institute of Kidney Diseases, West China Hospital of Sichuan University, Chengdu, China

**Keywords:** probiotic, synbiotic, chronic kidney disease, renal function, metabolism

## Abstract

**Background:**

Chronic kidney disease (CKD) is a serious and steadily growing health problem worldwide. Probiotic and synbiotic supplementation are expected to improve kidney function in CKD patients by altering imbalanced intestinal flora, regulating microbiota metabolites, modulating the brain-gut axis, and reducing inflammation.

**Objectives:**

Our aim is to report the latest and largest pooled analyses and evidence updates to explore whether probiotic and synbiotic have beneficial effects on renal function and general conditions in patients with CKD.

**Methods:**

We conducted a systematic literature search using PubMed, Embase, Web of Science, and the Cochrane Central Register of Controlled Trials from inception until 1 December 2023. Eligible literatures were screened according to inclusion and exclusion criteria, data were extracted, and a systematic review and meta-analysis was performed. Measurements included renal function-related markers, inflammatory markers, uremic toxins, lipid metabolism-related markers and electrolytes levels.

**Results:**

Twenty-one studies were included. The results showed that probiotic/synbiotic significantly reduced blood urea nitrogen (BUN) (standardized mean difference (SMD), −0.23, 95% confidence interval (CI) −0.41, −0.04; *p* = 0.02, I^2^ = 10%) and lowered c-reactive protein level (CRP) (SMD: −0.34; 95% CI: −0.62, −0.07; *p* = 0.01, I^2^ = 37%) in CKD patients, compared with the control group.

**Conclusion:**

In summary, probiotic/synbiotic supplementation seems to be effective in improving renal function indices and inflammation indices in CKD patients. Subgroup analyses suggested that longer-term supplementation is more favorable for CKD patients, but there is a high degree of heterogeneity in the results of partial subgroup analyses. The efficacy of probiotic/synbiotic in treating CKD needs to be supported by more evidence from large-scale clinical studies.

**Systematic review registration:**

https://www.crd.york.ac.uk/prospero/display_record.php?ID=CRD42024526836, Unique identifier: CRD42024526836.

## 1 Introduction

CKD is characterized by abnormalities in kidney structure or function that persist for a period of at least 3 months (1). The clinical features of CKD include decreased renal function and/or increased urinary albumin excretion (proteinuria) (2). It is a disease that progresses slowly over time, and a significant number of patients eventually reach end-stage renal disease and require dialysis for treatment (3, 4). Currently, the global prevalence of CKD is about 11%, and with increasing age, the prevalence in people over 70 years old is as high as 34% (5, 6). In addition, patients with CKD have an increased risk of cardiovascular disease, hypertension, diabetes, and infections (7). Although CKD has received considerable attention from scientists and clinicians, the care and treatment of the condition are still not up to par (8). Consequently, there is a pressing need to explore new drugs or therapeutic approaches.

Previous studies have shown that ecosystem imbalances are strongly associated with a number of chronic diseases, such as chronic kidney disease, diabetes and cardiovascular disease (9, 10). There is also evidence showed that imbalances in the gut microbiota may contribute to CKD (11). In addition, worsening CKD can further exacerbate imbalances in gut flora (12). Researchers proposed the gut-kidney axis and the CKD-colon axis in 2011 (13) and 2015 (14) to characterize the interactions between the kidney and the gut. Probiotics, synbiotic supplements are then expected to slow the progression of CKD by regulating the balance of intestinal flora (15). Probiotics, consisting of active microorganisms, colonize the human intestinal tract to improve the microbiological balance and benefit human health (16). Recent studies indicated that probiotics could potentially offer advantages to individuals with chronic kidney disease (17–19). The current definition of synbiotic has been updated to “a mixture of live microorganisms and substrates selectively utilized by host microorganisms to provide health benefits to the host” (20). There have been a number of studies aimed at evaluating the role of synbiotic supplementation in patients with CKD (21–24). Currently, non-food probiotics and synbiotic supplements are becoming increasingly available in the United States (25).

The use of probiotics has shown potential as a nutritional strategy for the prevention and/or treatment of CKD. In some animal studies, Lactobacillus supplementation has been shown to slow the progression of chronic kidney disease and delay renal failure by altering short-chain fatty acid and nicotinamide metabolism (26). In addition, an exploratory clinical study found that serum levels of tumor necrosis factor-α (TNF-α), Interleukin (IL)-6, IL-18, and endotoxin were significantly reduced in patients with CKD after probiotics administration (27). Despite growing interest in the potential role of probiotics in improving chronic kidney disease, there is a lack of extensive cross-sectional studies to comprehensively assess the effect of probiotics/synbiotics on the general condition of CKD patients in the population. In addition, although there have been previous meta-analyses of the relationship between probiotics and CKD, the outcome indicators of these analyses have focused on one of many metrics, such as kidney function or metabolism (28, 29). Therefore, a systematic review and meta-analysis incorporated latest RCT studies was designed to comprehensively investigate the effects of probiotics/synbiotics supplementation on renal function, lipid metabolism, inflammation, uremic toxin levels and electrolyte levels in dialysis/non-dialysis CKD patients.

## 2 Methods

### 2.1 Search strategy

The review program was established by two investigators (LC) and (WW) prior to the start of the study and registered with the PROSPERO International Prospective Registry of Systematic Reviews (registration number CRD42024526836). This study was conducted according to the Cochrane Manual and the Preferred Reporting Items for Systematic Reviews and Meta-Analyses (PRISMA) (30). Two independent reviewers (LC and YLT) searched PubMed, Embase, Web of Science and Cochrane Library from inception until December 2023. We searched the databases using the following terms: “probiotics,” “probiotic,” “synbiotics,” “synbiotic,” “renal insufficiency, chronic,” “chronic renal insufficiencies,” “renal insufficiencies, chronic,” “chronic renal insufficiency,” “kidney insufficiency, chronic,” “chronic kidney insufficiencies,” “kidney insufficiencies, chronic,” “chronic kidney diseases,” “chronic kidney disease” “disease, chronic kidney,” “diseases, chronic kidney,” “kidney disease, chronic,” “kidney diseases, chronic,” “chronic renal diseases,” “chronic renal disease,” “disease, chronic renal,” “diseases, chronic renal,” “renal disease, chronic,” “renal diseases, chronic” and “chronic kidney insufficiency.” Two researchers independently searched and evaluated the included studies, and any disagreements in the literature search were resolved by conferring with a third researcher (WW). Specific search strategies are shown in Supplementary Table 1.

### 2.2 Eligible criteria

The study met all of the following criteria (1) study design: randomized controlled study; (2) study participants: patients with a confirmed diagnosis of chronic kidney disease; (3) intervention: the intervention group should receive any dose of probiotic or synbiotic supplementation; (4) comparison regimen/control group: participants in the control group may receive a placebo or other medication and if other medications are used in the treatment group, they also control group must be used in the same way; (6) language: articles published in English.

Studies were excluded for the following reasons: (1) they were reviews, meta-analyses, case reports, conference abstracts, and guidelines; (2) the study was animal-based; (3) the study was published in a language other than English.

### 2.3 Research screening

**Figure 1:**
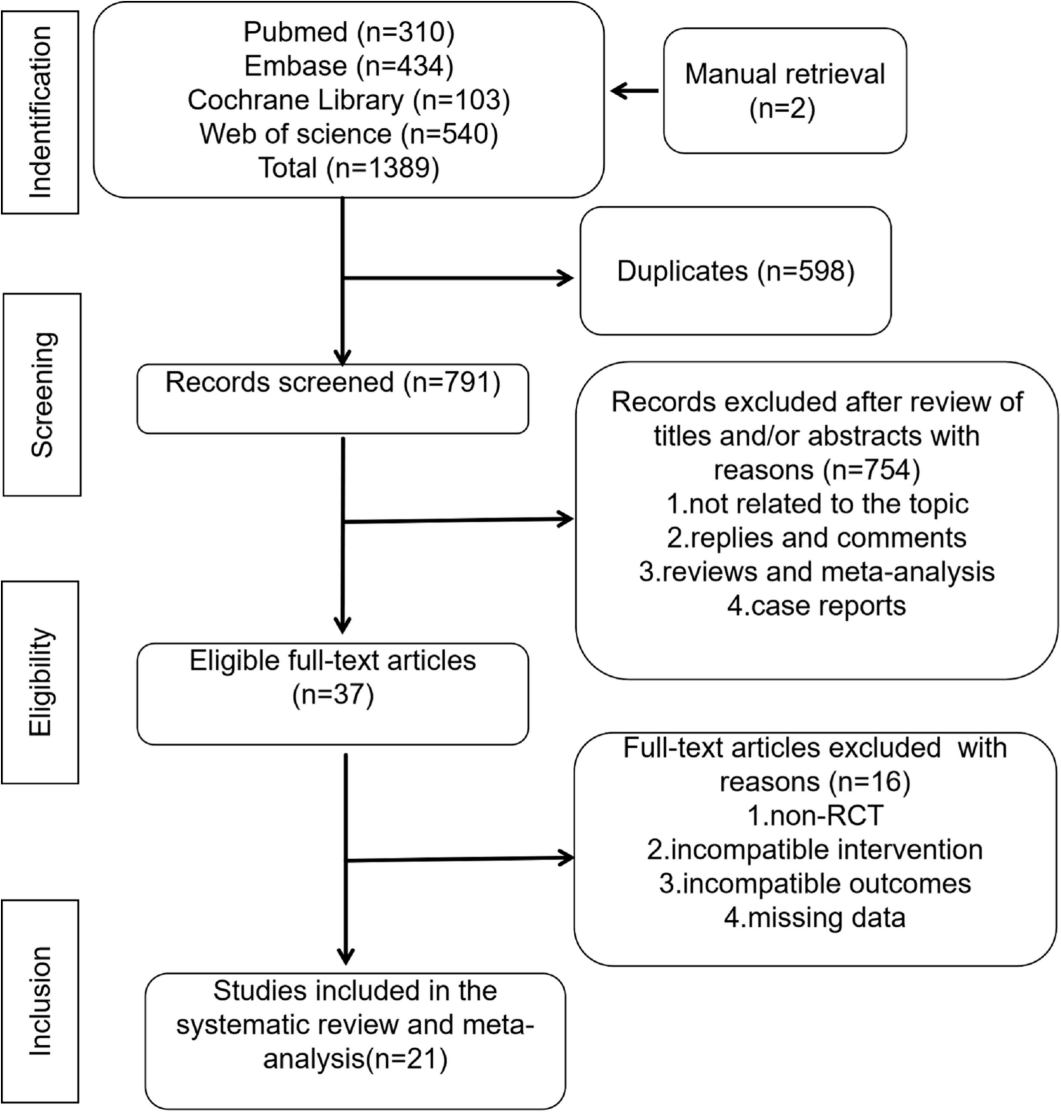
Flowchart of the employed literature search.

After excluding duplicate records, two researchers independently screened the titles and abstracts of all identified records to remove irrelevant documents. A full-text review was then conducted to determine eligibility for inclusion. Any disagreements regarding study selection could be resolved through discussion with a third researcher (LC, YLT, and WW). The study selection process is shown in Figure 1.

### 2.4 Data extraction

The following data were extracted from the included studies: (a) The basic information, including first author, publication year, region, data source, study design, and enrollment period; (b) Characteristics of the participants, including sample size, sex ratio (male), median age, median Body Mass Index (BMI) and hemodialysis time; (c) Interventions: probiotics/synbiotics types, dosage, frequency, intervention time; (d) Disease-related indicators: creatinine, BUN, eGFR, hemoglobin, uric acid, potassium, total cholesterol, HDL-cholesterol, LDL-cholesterol, indoxyl sulfate, p-cresyl sulfate, indole-3-acetic acid, CRP, IL-6, triglycerides, blood sodium, blood calcium and blood phosphorus. When continuous variables in the study were reported as median with range or interquartile range, we calculated the mean ± standard deviation through the validated mathematical method. When data were missing or not reported in the study, we contacted the corresponding authors to obtain completed data if available.

### 2.5 Quality assessment

Quality assessment of eligible RCTs was performed according to the Cochrane Handbook for Systematic Reviews of Interventions 5.1.0, based on seven terms: randomized sequence generation, allocation concealment, participant and personnel blinding, blinding of outcome assessment, incomplete outcome data, selective reporting and other biases sources (31). Three outcomes were assessed for each study, including low risk, high risk, and unclear risk. Studies with more “low risk” of bias assessment were considered superior.

### 2.6 Statistical analysis

Evidence synthesis was performed in Review Manager 5.4 version (Cochrane Collaboration, Oxford, United Kingdom). The SMD was applied for the comparison of continuous variables. All metrics were reported with mean ± SD. The heterogeneity in studies was assessed through the inconsistency index (I^2^). I^2^ > 50% were considered as significant heterogeneity. A random-effect model was used to estimate the combined SMD when significant heterogeneity was detected (I^2^ > 50%). Otherwise, the fixed-effect model was applied. In addition, we performed one-way sensitivity analyses to evaluate the effect of included studies on the combined results for outcomes with significant heterogeneity. Subgroup analyses were used to explore sources of heterogeneity. The subgroup analysis has not been conducted for indicators which were few included in literatures. Because the limited number of literatures may lead to a significant discrepancy between subgroups, which could impact the accuracy of the results. Publication bias was evaluated visually by creating funnel plots via Review Manager 5.4 version (Cochrane Collaboration, Oxford, United Kingdom), as well as by conducting Egger’s regression tests using Stata 15.0 version (Stata Corp, College Station, TX, United States) for outcomes with 5 or more included studies. *p*-value < 0.05 was considered as statistically significant publication bias.

## 3 Results

### 3.1 Literature search

The initial search was completed on 1 December 2023. We have identified 310 potentially relevant publications from PubMed, 434 from Embase, 103 from The Cochrane library, 540 from Web of science and 2 from manual retrieval. Endnote was used to eliminate duplicate publications, resulting in 791 records for review. After excluding publications that did not meet the inclusion criteria, we included 21 studies for systematic review and meta-analysis. A flow diagram illustrating the exclusion of articles with specific reasons is shown in Figure 1 (PRISMA flowchart).

### 3.2 Study characteristics

We conducted a systematic review and meta-analysis of 869 patients with chronic kidney disease involved in 21 RCT studies (17–19, 21, 22, 32–47). The sample size ranged between 11 and 80, and the mean age of the patients was recorded ranged from a minimum of 45 years old to a maximum of 76 years old. The majority of the patients were from Asia. The articles were studied from 2013 to 2023 (Table 1).

**Table 1:** Baseline characteristics of include studies and methodological assessment.

Authors	Study period	Country	Study design	Patients (*n*)	male (*n*)	Intervention	Median follow-up (months)	Comparator	Average age (years)	Mean BMI (kg/m^2^)
				Intervention/control	Intervention/control				Intervention/control	Intervention/control
Carmela Cosola et al. (21)	2021	Italy	RCT	13/10	7/7	Synbiotic (Lactobacillus casei LC4P1 2.4 × 10^9^, Bifidobacterium animalis BLC1 2.4 × 10^9^), prebiotics (fructoligosaccharides 2.5 g and inulin 2.5 g) and natural antioxidants (a mix of quercetin 0.064 g, resveratrol 0.023 g and proanthocyanidins 0.013 g) (2 bags/day)	2	Placebo	51.0/51.5	27.2/25.8
Maria Teresa Rocchetti et al. (22)	2020	Italy	RCT	6/5	5/4	Synbiotic consisted of probiotics (Lactobacillus casei LC4P1 3.2 × 10^9^, Lactobacillus bulgaricus SP5 3.2 × 10^9^, and Bifidobacterium animalis BLC1 3.2 × 10^9^), prebiotics (fructoligosaccharides 5 g and inulin 5 g), natural antioxidants (a mix of quercetin 0.13 g, resveratrol 46 μg, and proanthocyanidins 25 μg)	2	Placebo	56.0/52.0	−/−
Banos, Amanda de Faria et al. (32)	2018	Brazil	RCT	12/10	6/5	Streptococcus thermophilus, Lactobacillus acidophilus, and Bifidobacteria longum (total 90 billion CFU/day)	3	Placebo	64.7/63.6	26.7/27.2
Borges et al. (33)	2017	Brazil	RCT	16/17	11/10	Streptococcus thermophilus, Lactobacillus acidophilus, and Bifidobacteria longum (total 90 billion CFU/day)	3	Placebo	53.6/50.3	25.3/25.2
Farzad Eidi et al. (34)	2018	Iran	RCT	21/21	15/17	Lactobacillus rhamnosus (1.6*10^7^ CFU/day)	1	Placebo	57.0/59.6	24.1/24.7
Guida et al. (35)	2014	Italy	RCT	18/12	14/12	5*10^9^ Lactobacillus plantarum, 2*10^9^ Lactobacillus casei subsp. Rhamnosus, 2*10^9^ Lactobacillus gasseri, 1*10^9^ Bifidobacterium infantis, 1*10^9^ Bifidobacterium longum, 1*10^9^ Lactobacillus acidophilus, 1*10^9^ Lactobacillus salivarius, 1*10^9^ Lactobacillus sporogenes, 5*10^9^ Streptococcus thermophilus (5 g/day)	1	Placebo	57.0/63.2	26.4/28.4
Neda Haghighat et al. (36)	2018	Iran	RCT	23/19	12/10	probiotics powder containing Lactobacillus acidophilus strain T16, Bifidobacterium bifidum strain BIA-6, Bifidobacterium lactis strain BIA-6, and Bifidobacterium longum strain LAF-5 (2.7 × 10^7^ CFU/g each) (5 g/day)	3	Placebo	48.0/45.4	23.8/23.3
Michael Laffin et al. (37)	2019	Canada	RCT	9/11	6/7	HAM-RS2 (the first month: 20 g/d, the second month: 25 g/d)	2	Placebo	46.2/45.4	−/−
Priya Lakshmi et al. (19)	2020	India	RCT	40/40	−/−	Lobun Probiotics (twice/day)	3	Placebo	−/−	−/−
Paik Seong Lim et al. (17)	2021	China	RCT	25/25	10/10	Lactococcus lactis subsp. Lactis LL358, Lactobaccillus salivarius LS159, Lactobaccillus pentosus LPE588 (1 * 10^11^CFU/day)	6	Placebo	53.8/57.6	24.2/24.8
Aida Lydia et al. (38)	2022	Indonesia	RCT	27/30	10/11	Capsules containing synbiotics (Lactobacillus acidophilus and Bifdobacterium longum 5×10^9^ CFU and 60 mg of fructooligosaccharides; two capsules/day)	2	Placebo	51.2/52.3	22.5/24.2
Catherine McFarlane et al. (39)	2017–2018	Australia	RCT	35/33	23/22	high resistant starch fiber supplement (20 g/day); probiotic component of nine different strains from three different genera (Bifidobacteria, Lactobacillus, and Streptococcus) (4.5 × 10^11^ CFU/day)	12	Placebo	71.2/65.8	−/−
Soheila Mirzaeian et al. (40)	2015–2016	Iran	RCT	21/21	14/16	Capsules containing 500 mg symbiotic: Lactobacillus casei (3.5 * 10^9^ CFU), L. acidophilus (1.5*10^9^ CFU), L. rhamnosus (3 *10^9^ CFU), L. bulgaricus (3.5* 10^8^ CFU), Bifidobacterium breve (5 * 10^9^ CFU), B. longum (1 * 10^10^ CFU), and Streptococcus thermophiles (3.5*10^8^ CFU) (two capsules/day)	2	Placebo	58.3/69.7	24.8/24.6
Milos Mitrovic, MD et al. (41)	2019–2020	Serbia	RCT	17/17	9/9	Pills containing 16 billion colonies of Lactobacillus acidophilus CBT LA1 (4 * 10^9^), Lactobacillus casei CBT LC5 (4 * 10^9^), and Bifidobacterium lactis CBT BL3 (8 * 10^9^) (2 pills/day); 1.6 g of inulin	3	Placebo	69/69	26.5/25.5
Anita Saxena et al. (42)	2019	Multicenter	RCT	29/21	−/−	Pills containing probiotics (Lactobacillusacidophillus 100 mg, Bifidobacteriumlongumm 100 mg, Streptococcus thermophilus 50 mg), prebiotics (FOS 100 mg), and proteolytic enzymes (150 mg with an activity 355,000 IU) (three times/day)	3	Placebo	54/49	21.6/26.0
Zahra Shariaty et al. (18)	2014	Iran	RCT	17/17	10/10	L. acidophilus (3 × 10^10^ CFU/g), Lactobacillus casei (3 × 10^9^ CFU/g), Lactobacillus rhamnosus (7 × 10^9^ CFU/g), Lactobacillus bulgaricus (5 × 10^8^ CFU/g), Bifidobacterium breve (2 × 10^10^ CFU/g), Bifidobacterium longum (1 × 10^9^ CFU/g), S. thermophilus (3 × 10^8^ CFU/g) (total 500 mg/day)	3	Placebo	54.1/61.5	−/−
Alireza Soleimani et al. (43)	2017–2018	Iran	RCT	30/30	21/21	Synbiotic capsule containing Lactobacillus acidophilus, Lactobacillus casei, and Bifidobacterium bifidum (2 × 10^9^ CFU/day each); 0.8 g/day of inulin	3	Placebo	62.8/62.8	26.4/26.9
Rita de Cássia Stampini Oliveira Lopes et al. (44)	2018	Brazil	RCT	29/29	17/21	100 mL of probiotic dairy drink and 40 g of extruded sorghum flakes, probiotic bacterium Bifidobacterium longum BL-G301 (7.4 × 10^8^ ± 5.4 × 10^8^ CFU/100 mL)	1.75	Placebo	63.1/63.0	−/−
Hamid Tayebi-Khosroshahi et al. (45)	2016	Iran	RCT	16/16	9/5	30 mm lactulose syrup (three times/ day)	2	Placebo	59.3/56.8	−/−
Francisco Hevilla et al. (46)	2023	Multicenter	RCT	11/10	7/8	Probiotics [Each capsule (380 g)] containing: Bifidobacterium breve CNCM I-4035 (1.00E + 09 CFU), Bifidobacterium animalis lactis BPL1 CECT 8145 (3.50E + 09 CFU), and Lactobacillus paracasei CNCM I-4034 (5.00E + 08 CFU); dietary recommendations	6	Individualized dietary recommendations	66/76.3	−/−
Alireza Mafi et al. (47)	2018	Iran	RCT	30/30	−/−	Probiotic supplements containing Lactobacillus acidophilus strain ZT-L1, Bifidobacterium bifidum strain ZT-B1, Lactobacillus reuteri strain ZT-Lre, and Lactobacillus fermentum strain ZT-L3 (each 2 × 10^9^) (8 × 10^9^ CFU/day)	3	Placebo	58.9/60.9	25.3/26.3

### 3.3 Risk of bias assessment

The risk of bias assessment is presented in Supplementary Figures S1, S2. Most of the included studies were considered to have a low or unclear risk of bias. Two papers had significant baseline imbalance (17, 36). The main source of bias in two pieces of literatures was the failure to implement double blinding (21, 44). Six literatures were at high risk because of poor completeness of data results (19, 32–34, 44, 46).

### 3.4 Renal function parameters

#### 3.4.1 Change in blood urea nitrogen

**Figure 2:**
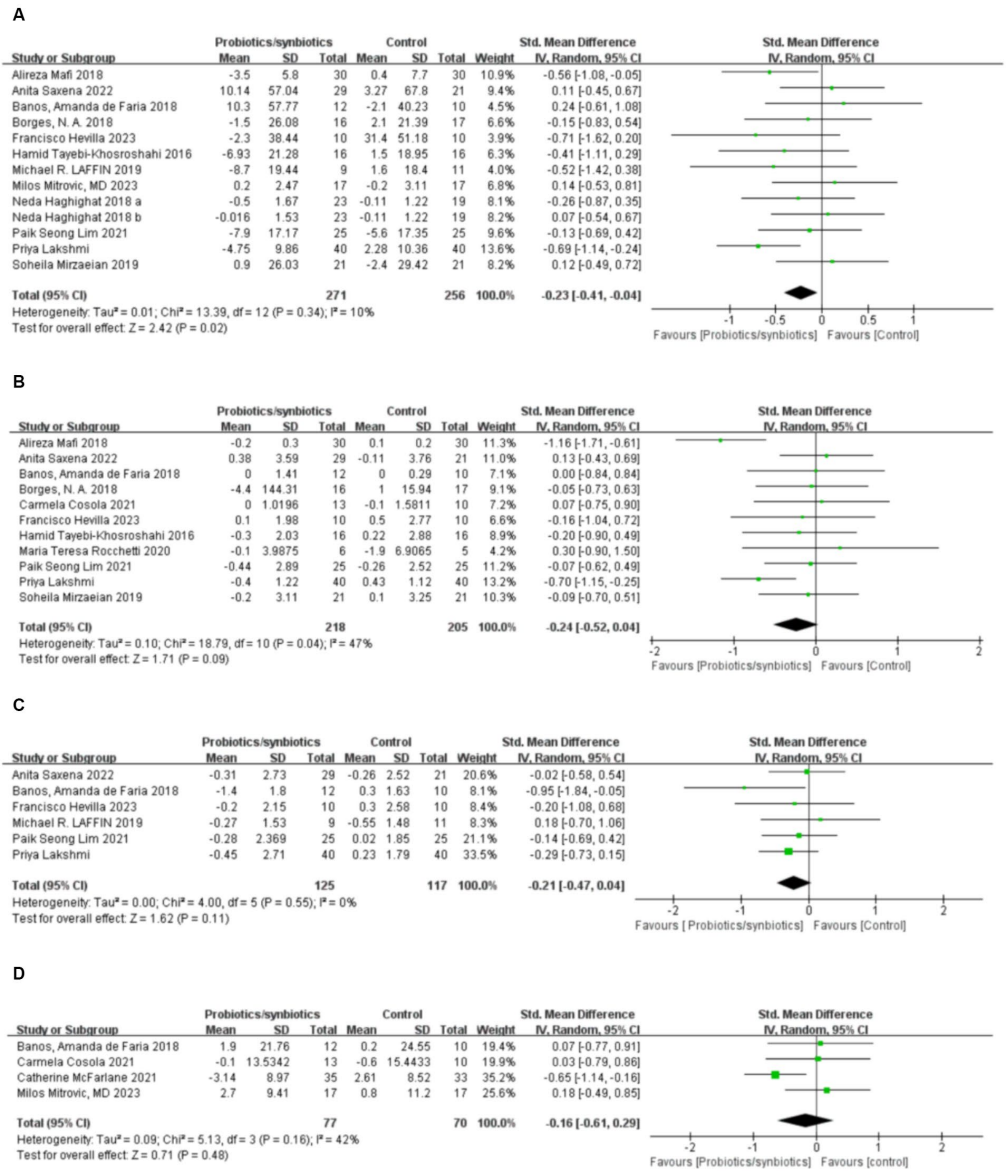
Forest plots of kidney function outcomes: **(A)** BUN, **(B)** serum creatinine, **(C)** uric acid, **(D)** eGFR.

**Figure 3:**
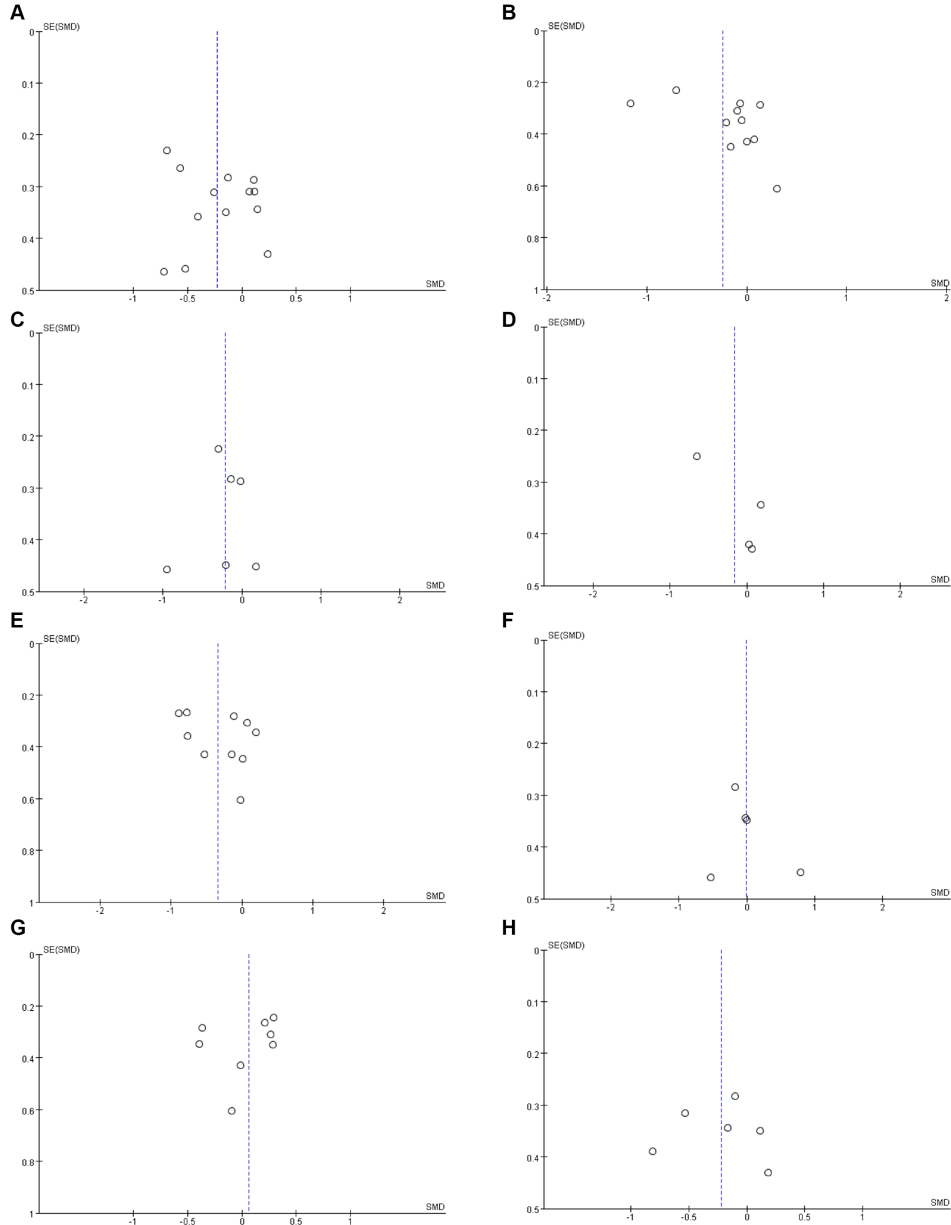
Funnel plots of **(A)** BUN, **(B)** serum creatinine, **(C)** uric acid, **(D)** eGFR, **(E)** CRP, **(F)** IL-6, **(G)** indoxyl sulfate **(H)** p-cresyl sulfate.

Twelve studies (17, 19, 32, 33, 36, 37, 40–42, 45–47) including 527 patients (271 probiotics/synbiotics; 256 controls) were included in the evaluation of blood urea nitrogen (BUN). Pooled analysis showed that the reduction of BUN in patients treated with probiotics/synbiotics was significantly better than in the control group (SMD: −0.23; 95% CI: −0.41, −0.04; *p* = 0.02; Figure 2A). No evidence of significant heterogeneity (I^2^ = 10%, *p* = 0.34) and statistical (Egger’s test, *p* = 0.452) or visual (Figure 3A) publication bias was detected.

Based on a range of subgroup analyses, we did not observe an effect of probiotic/synbiotics supplementation on BUN in American patients (k = 3, SMD: −0.13, 95% CI: −0.59, 0.33, I^2^ = 0%, *p* = 0.57). However, we found a significant decrease in BUN following probiotic/synbiotics supplementation in Asian individuals (k = 8, SMD: −0.28, 95% CI: −0.48, −0.09, I^2^ = 22%, *p* = 0.004). In addition, probiotic/synbiotics supplementation significantly reduced BUN in the long-term treatment (≥3 months; k = 10, SMD: −0.23, 95% CI: −0.44, −0.01, I^2^ = 21%, *p* = 0.04), but not in the short term (<3 months; k = 3, SMD: −0.19, 95% CI: −0.60, 0.22, I^2^ = 0%, *p* = 0.35). In addition, probiotics/synbiotics did not change BUN level in the HD patients (k = 7, SMD: −0.15, 95% CI: −0.4, 0.1, I^2^ = 0%, *p* = 0.23). However, probiotics/synbiotics significantly decreased the BUN level in non-HD patients (k = 6, SMD: −0.31, 95% CI: −0.54, −0.07, I^2^ = 45%, *p* = 0.01). And probiotics/synbiotics did not change BUN level in patients ≥60 years (k = 4, SMD: 0.01, 95% CI: −0.35, 0.38, I^2^ = 0%, *p* = 0.95) and patients <6 0 y (k = 8, SMD: −0.21, 95% CI: −0.43, 0.00, I^2^ = 0%, *p* = 0.05; Table 2).

**Table 2:** Subgroup analyses.

Subgroup	Change in BUN				Change in serum creatinine				Change in indoxyl sulfate				Change in CRP				Change of blood phosphorus			
	Study	SMD [95%CI]	*P*-value	*I* ^2^	Study	SMD [95%CI]	*P*-value	*I* ^2^	Study	SMD [95%CI]	*P*-value	*I* ^2^	Study	SMD [95%CI]	*P*-value	*I* ^2^	Study	SMD [95%CI]	*P*-value	*I* ^2^
Total	13	−0.23 [−0.41, −0.04]	0.02	10%	11	−0.24 [−0.52, 0.04]	0.09	47%	8	0.06 [−0.16, 0.28]	0.58	0%	10	−0.34 [−0.62, −0.07]	0.01	37%	10	−0.08 [−0.28, 0.11]	0.41	0%
**Patient population**																				
Hemodialysis	7	−0.15 [−0.40, 0.10]	0.23	0%	5	−0.06 [−0.37, 0.26]	0.72	0%	4	0.03 [−0.29, 0.34]	0.87	0%	5	−0.16 [−0.49, 0.17]	0.34	29%	7	−0.05 [−0.29, 0.20]	0.72	0%
Non-hemodialysis	6	−0.31 [−0.54, −0.07]	0.01	45%	6	−0.36 [−0.80, 0.09]	0.11	66%	4	0.10 [−0.21, 0.42]	0.53	0%	5	−0.67 [−1.02, −0.33]	0.0001	0%	3	−0.14 [−0.46, 0.18]	0.38	0%
**Region**																				
Asia	8	−0.28 [−0.48, −0.09]	0.004	22%	6	−0.36 [−0.76, 0.04]	0.07	67%	4	0.03 [−0.36, 0.42]	0.86	31%	5	−0.33 [−0.76, 0.11]	0.14	65%	8	−0.08 [−0.29, 0.14]	0.48	0%
Europe	1	0.14 [−0.53, 0.81]	0.69	NA	2	0.15 [−0.53, 0.83]	0.67	0%	2	−0.32 [−0.91, 0.27]	0.29	0%	3	−0.56[−1.05, −0.07]	0.03	0%	1	0.00 [−0.82, 0.82]	1	0%
America	3	−0.13 [−0.59, 0.33]	0.57	0%	2	−0.03[−0.56, 0.5]	0.03	61%	2	0.17[−0.37, 0.70]	0.54	0%	1	−0.15 [−0.99, 0.69]	0.73	NA	1	0.07 [−0.81, 0.96]	0.87	NA
**Treatment time**																				
≥3 months	10	−0.23[−0.44, −0.01]	0.04	21%	7	−0.32[−0.7, 0.06]	0.1	63%	5	−0.02[−0.33, 0.29]	0.90	19%	6	−0.51[−0.83, −0.19]	0.002	33%	6	−0.16[−0.39, 0.08]	0.19	0%
<3 months	3	−0.19[−0.60, 0.22]	0.35	0%	4	−0.05[−0.43, 0.33]	0.79	0%	3	0.20 [−0.18, 0.58]	0.30	0%	4	−0.02[−0.40, 0.36]	0.92	0%	4	0.09[−0.26, 0.44]	0.62	0%
**Mean/median age**																				
≥60 years	4	0.01[−0.35, 0.38]	0.95	0%	3	−0.08[−0.51, 0.34]	0.70	0%	4	0.11[−0.20, 0.41]	0.50	10%	5	−0.36[−0.76, 0.03]	0.07	39%	2	−0.04[−0.57, 0.48]	0.87	7%
<60 years	8	−0.21[−0.43, 0.00]	0.05	0%	7	−0.28[−0.64, 0.08]	0.13	60%	4	0.02[−0.31, 0.34]	0.93	0%	5	−0.31[−0.75, 0.12]	0.16	48%	7	−0.03[−0.27, 0.22]	0.84	0%

OR, odds ratio; CI, confidence interval.

#### 3.4.2 Change in serum creatinine

Eleven articles (17, 19, 21, 22, 32, 33, 40, 42, 45–47) were included in the analysis of serum creatinine levels involving 423 patients (218 probiotics/synbiotics; 205 controls). The evidence synthesis showed similar changes of serum creatinine in patients in the probiotic/synbiotics group and the placebo group (SMD: −0.24; 95% CI: −0.52, 0.04; *p* = 0.09) without significant heterogeneity (I^2^ = 47%, *p* = 0.04; Figure 2B). No publication bias was detected by the funnel plot (Figure 3B) or Egger’s test (*p* = 0.097).

Based on a series of subgroup analyses (Table 2), we did not observe any changes in serum creatinine following probiotic/synbiotics supplementation in individuals on hemodialysis (k = 5, SMD: −0.06, 95% CI: −0.37, 0.26, I^2^ = 0%, *p* = 0.72) and non-hemodialysis individuals (k = 6, SMD: −0.36, 95% CI: −0.8, 0.09, I ^2^ = 66%, *p* = 0.11). Based on geographical location, we observed no significant change of serum creatinine in countries located in America (k = 2; SMD: −0.03, 95% CI: −0.56, 0.5, I^2^ = 61%, *p* = 0.03), Asia (k = 6, SMD: −0.36, 95% CI: −0.76, 0.04, I^2^ = 67%, *p* = 0.07) and Europe (k = 2; SMD: 0.15, 95% CI: −0.53, 0.83, I^2^ = 0%, *p* = 0.67). In addition, probiotics/synbiotics did not change the serum creatinine in the short term (<3 months; k = 4, SMD: −0.05, 95% CI: −0.43, 0.33, I^2^ = 0%, *p* = 0.79) and the long term (≥3 months; k = 7, SMD: −0.32, 95% CI: −0.7, 0.06, I^2^ = 63%, *p* = 0.1) or in older patients ≥ 60 years (k = 3, SMD: −0.08, 95% CI: −0.51, 0.34, I^2^ = 0%, *p* = 0.7) and younger patients < 60 years (k = 7, SMD: −0.28, 95% CI: −0.64, 0.08, I^2^ = 60%, *p* = 0.13).

#### 3.4.3 Change in uric acid

Six studies (17, 19, 32, 37, 42, 46) including 242 patients (125 probiotics/synbiotics; 117 controls) were included in the analysis of uric acid. Pooled analysis showed similar levels of alteration of uric acid in the probiotic/synbiotics group and the control group (SMD: −0.21; 95% CI: −0.47, 0.04; *p* = 0.11; Figure 2C). No significant heterogeneity (I^2^ = 0%, *p* = 0.55) and no evidence of statistical (Egger’s test, *p* = 0.799) or visual (Figure 3C) publication bias was observed.

#### 3.4.4 Change in eGFR

Four articles (21, 32, 39, 41) reported data on eGFR levels between the two groups of 147 cases (77 probiotics/synbiotics; 70 controls). Evidence synthesis observed similar changes in eGFR levels in patients with probiotics/synbiotics and placebo (SMD: −0.16; 95% CI: −0.61, 0.29; *p* = 0.48), with significant heterogeneity (I^2^ = 42%, *p* = 0.16; Figure 2D). No evidence of visual publication bias was observed (Figure 3D).

### 3.5 Inflammation indicators and uremic toxins

#### 3.5.1 Change in C-reactive protein

**Figure 4:**
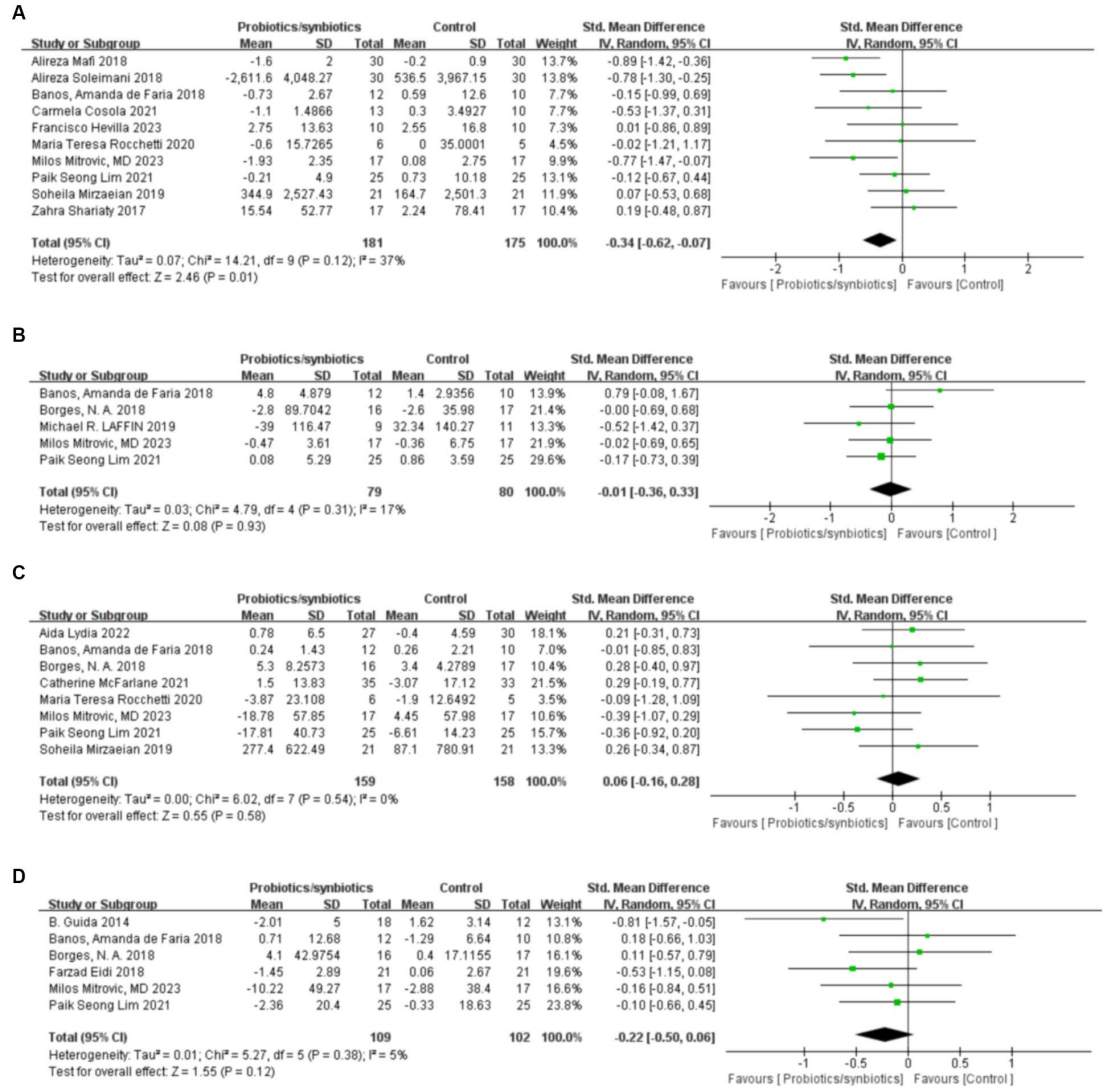
Forest plots of inflammation and uremic toxins outcomes: **(A)** CRP, **(B)** IL-6, **(C)** Indoxyl sulfate, **(D)** p-cresyl sulfate.

Ten studies (17, 18, 21, 22, 32, 40, 41, 43, 46, 47) with a total of 356 patients (181 probiotics/synbiotics patients; 175 control patients) were included in the analysis of CRP. Pooled analysis showed that the probiotic/synbiotics group was significantly more effective in reducing CRP than the control group (SMD: −0.34; 95% CI: −0.62, −0.07; *p* = 0.01; Figure 4A). No significant heterogeneity was observed (I^2^ = 37%, *p* = 0.12) and no evidence of statistical (Egger’s test, *p* = 0.288) or visual (Figure 3E) publication bias was observed.

Subgroup analysis based on patient population suggested no changes in CRP following probiotics/synbiotics supplementation in individuals on hemodialysis (k = 5, SMD: −0.16, 95% CI: −0.49, 0.17, I^2^ = 29%, *p* = 0.34; Table 2). Furthermore, probiotics/synbiotics did not change CRP level in American individuals (k = 5, SMD: −0.33, 95% CI: −0.76, 0.11, I^2^ = 65%, *p* = 0.14), older individuals (k = 5, SMD: −0.36, 95% CI: −0.76,0.03, I^2^ = 39%, *p* = 0.07), younger individuals (k = 5, SMD: −0.31, 95% CI: −0.75, 0.12, I^2^ = 48%, *p* = 0.16) and individuals treated for a shorter period of time (k = 4, SMD: −0.02, 95% CI: −0.40, 0.36, I^2^ = 0%, *p* = 0.92). However, we found a significant decrease in CRP following probiotics/synbiotics supplementation in non-hemodialysis individuals (k = 5, SMD: −0.67, 95% CI: −1.02, −0.33, I^2^ = 0%, *p* = 0.0001), individuals treated for a longer period of time (k = 4, SMD: −0.51, 95% CI: −0.83, −0.19, I^2^ = 33%, *p* = 0.002) and European individuals (k = 3, SMD: −0.56, 95% CI: −1.05, −0.07, I^2^ = 0%, *p* = 0.03).

#### 3.5.2 Change in IL-6

Five studies (17, 32, 33, 37, 41) were included in the analysis of IL-6, comprising a total of 159 patients (79 probiotics/synbiotics patients; 80 control patients). Pooled analysis suggested no statistically significant difference in the change of IL-6 levels between the two groups (SMD: −0.01; 95% CI: −0.36, 0.33; *p* = 0.93; Figure 4B). No significant heterogeneity was observed (I^2^ = 17%, *p* = 0.31). Nor was evidence of publication bias observed statistically Egger’s test, (*p* = 0.626) or visually (Figure 3F).

#### 3.5.3 Change in indoxyl sulfate

Three hundred and seventeen patients from 8 studies (17, 22, 32, 33, 38–41) were included in the analysis of indoxyl sulfate (159 probiotics/synbiotics patients; 158 control patients). No statistically significant differences were found in the results of the pooled analysis between the two groups (SMD: 0.06; 95% CI: −0.16, 0.28; *p* = 0.58; Figure 4C). No significant heterogeneity was observed (I^2^ = 0%, *p* = 0.54) as well as evidence of statistical (Egger’s test, *p* = 0.507) or visual (Figure 3G) publication bias.

Subgroup analysis based on patient population revealed no changes in indoxyl sulfate level after evaluation with either hemodialysis patients (k = 4, SMD: 0.03, 95% CI: −0.29, 0.34, I^2^ = 0%, *p* = 0.87) or non-hemodialysis patients (k = 4, SMD: 0.10, 95% CI: −0.21, 0.42, I^2^ = 0%, *p* = 0.53; Table 2). Furthermore, probiotics did not change the indoxyl sulfate level in the short term (<3 months; k = 3, SMD: 0.20, 95% CI: −0.18, 0.58, I^2^ = 0%, *p* = 0.30) and the long term (≥3 months; k = 5, SMD: −0.02, 95% CI: −0.33, 0.29, I^2^ = 19%, *p* = 0.90) or in older patients (≥60 years; k = 4, SMD: 0.11, 95% CI: −0.20, 0.41, I^2^ = 10%, *p* = 0.50) and younger patients (<60 years; k = 4, SMD: 0.02, 95% CI: −0.31, 0.34, I^2^ = 0%, *p* = 0.93). Besides, probiotics did not change the indoxyl sulfate level in Asia individuals (k = 4, SMD: 0.03, 95% CI: −0.36, 0.42, I^2^ = 31%, *p* = 0.86), America individuals (k = 2, SMD: 0.17, 95% CI: −0.37, 0.70, I^2^ = 0%, *p* = 0.54) and European individuals (k = 2, SMD: −0.32, 95% CI: −0.91, 0.27, I^2^ = 0%, *p* = 0.29).

#### 3.5.4 Change in p-cresyl sulfate

A total of 211 patients from 6 studies (17, 32–35, 41) were included in the analysis of p-cresyl sulfate (109 probiotics/synbiotics patients; 102 control patients). No significant statistical differences were found in the results of the pooled analyses between the probiotics/synbiotics group and the control group (SMD: −0.22; 95% CI: −0.5, 0.06; *p* = 0.12; Figure 4D). No significant heterogeneity (I^2^ = 5%, *p* = 0.38) was observed as well as statistical (Egger’s test, *p* = 0.947) or visual (Figure 3H) evidence of publication bias.

### 3.6 Lipid metabolism-related indicators

#### 3.6.1 Change in total cholesterol

**Figure 5:**
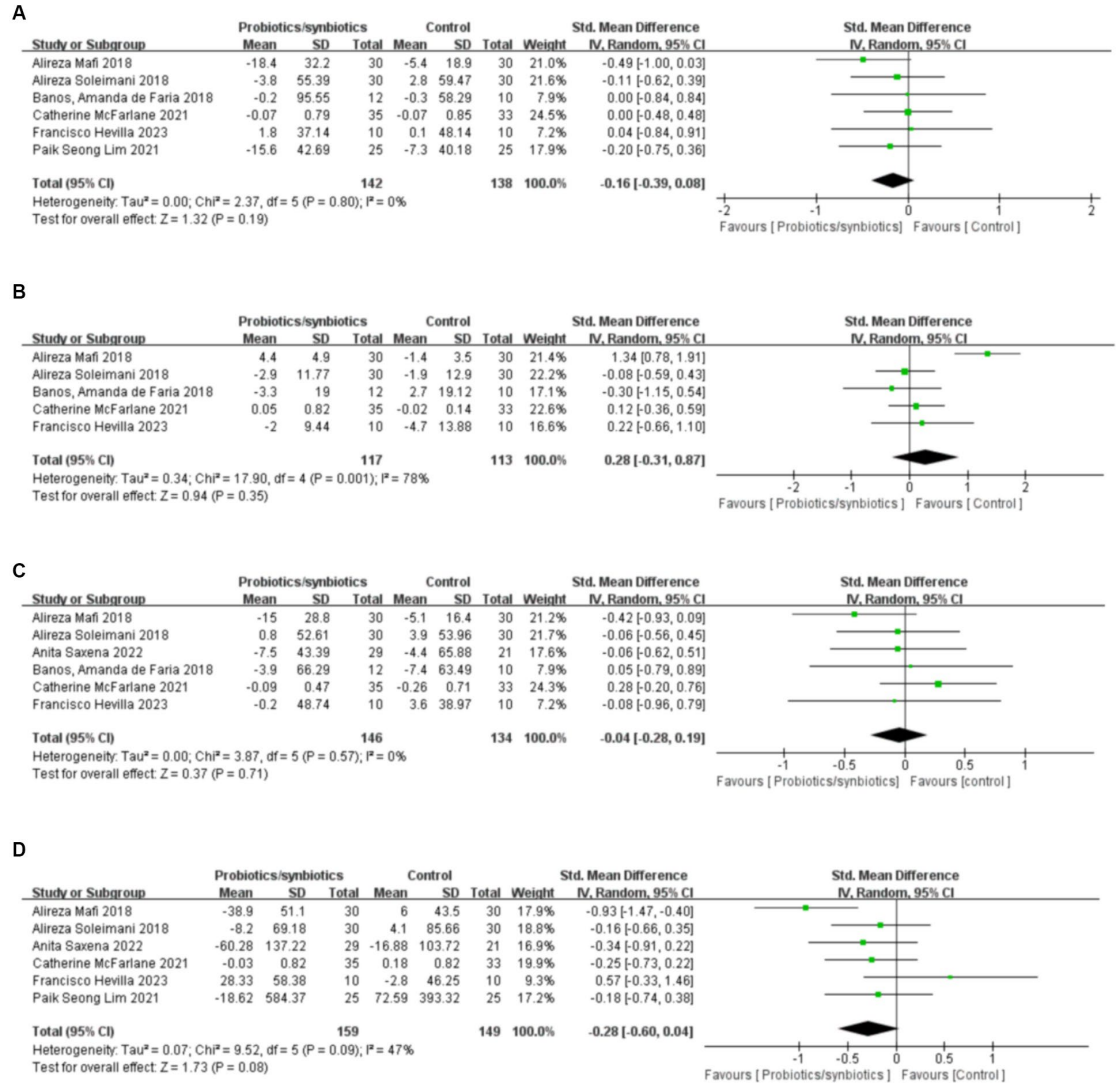
Forest plots of lipid metabolism evaluation outcomes: **(A)** total cholesterol, **(B)** HDL, **(C)** LDL, **(D)** triglyceride.

**Figure 6:**
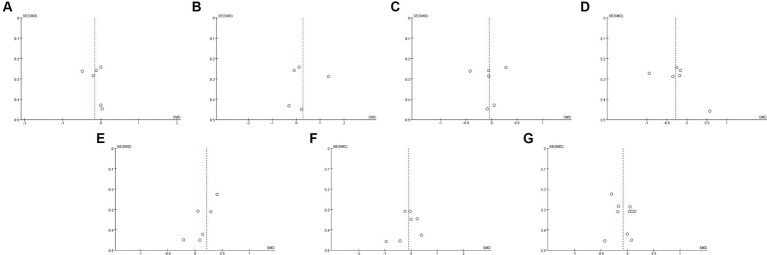
Funnel plots of **(A)** total cholesterol, **(B)** HDL, **(C)** LDL, **(D)** triglyceride, **(E)** blood calcium, **(F)** blood potassium, **(G)** blood phosphorus.

A total of six articles (17, 32, 39, 43, 46, 47) involving 280 patients were included to analyze total cholesterol levels (142 probiotics/synbiotics patients; 138 control patients). Pooled analysis showed that the changes in total cholesterol levels were similar in the probiotics/synbiotics group and the control group (SMD: −0.16; 95% CI: −0.39, 0.08; *p* = 0.19) with no significant heterogeneity (I^2^ = 0%, *p* = 0.8; Figure 5A). Neither the funnel plot (Figure 6A) nor the Egger test (*p* = 0.544) revealed publication bias.

#### 3.6.2 Change in high density lipoprotein

Five studies (32, 39, 43, 46, 47) were included in the analysis of high density lipoprotein (HDL), involving 230 patients (117 probiotics/synbiotics patients; 113 control patients). No significant statistical differences were found in the results of the pooled analyses between the probiotics/synbiotics group and the control group (SMD: 0.28; 95% CI: −0.31, 0.87; *p* = 0.35; Figure 5B). Significant heterogeneity (I^2^ = 78%, *p* = 0.001) was observed. There was no statistical (Egger’s test, *p* = 0.874) or visual (Figure 6B) evidence of publication bias.

#### 3.6.3 Change in low density lipoprotein

Six articles (32, 39, 42, 43, 46, 47) were included in the analysis for low density lipoprotein (LDL), involving 280 patients (146 probiotics/synbiotics group patients; 134 control patients). Evidence synthesis showed that probiotics/synbiotics group had a similar change in LDL level with the control group (SMD: −0.04; 95% CI: −0.28, 0.19; *p* = 0.71) with no significant heterogeneity (I ^2^ = 0%, *p* = 0.57; Figure 5C). Both funnel plot (Figure 6C) and Egger’s test (*p* = 0.936) did not detect publication bias.

#### 3.6.4 Change in triglyceride

Analysis of triglyceride levels in 308 patients (159 probiotic/synbiotics patients; 149 control patients) from six publications (17, 39, 42, 43, 46, 47) showed that there was no statistically significant difference (SMD: −0.28; 95% CI: −0.6, 0.04; *p* = 0.08) or significant heterogeneity (I ^2^ = 47%, *p* = 0.09) in the change of triglyceride levels in the probiotics/synbiotics group compared with the control group (Figure 5D). No publication bias was found after both funnel plot (Figure 6D) and Egger’s test (*p* = 0.26) evaluation.

### 3.7 Electrolytes

#### 3.7.1 Change in blood calcium

**Figure 7:**
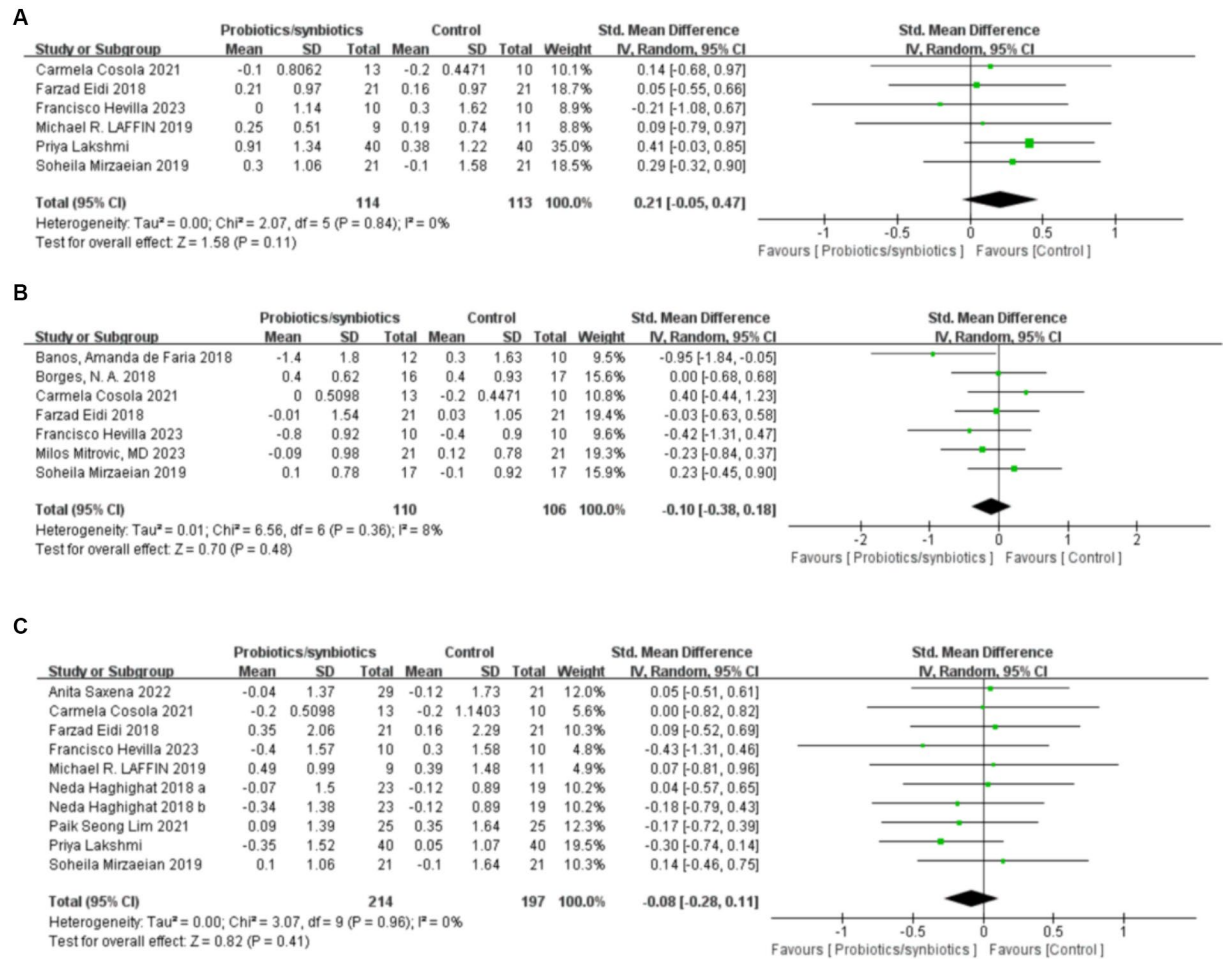
Forest plots of electrolytic outcomes: **(A)** blood calcium, **(B)** blood potassium, **(C)** blood phosphorus.

Six publications (19, 21, 34, 37, 40, 46) involving 227 patients (114 probiotics/synbiotics patients; 113 control patients) were included in the analysis regarding blood calcium levels, and the results suggested that there were no statistically significant differences (SMD: 0.21; 95% CI: −0.05, 0.47; *p* = 0.11) and no significant heterogeneity (I ^2^ = 0%, *p* = 0.84) in the change of blood calcium levels in patients in the probiotics/synbiotics group compared with the control group (Figure 7A). It is worth noting that funnel plots (Figure 6E) and Egger’s test revealed significant publication bias (*p* = 0.049).

#### 3.7.2 Change in blood potassium

Two hundred and sixteen patients (110 probiotics/synbiotics patients; 106 control patients) originating from seven publications (21, 32–34, 40, 41, 46) were included in the analysis regarding blood potassium levels. And the results suggested that there were no statistically significant differences (SMD: −0.1; 95% CI: −0.38, 0.18; *p* = 0.48) and no significant heterogeneity (I ^2^ = 8%, *p* = 0.36) in the change of blood potassium levels of the patients in the probiotics/synbiotics group compared with the control group (Figure 7B). Funnel plot (Figure 6F) and Egger’s test did not reveal significant publication bias (*p* = 0.426).

#### 3.7.3 Change in blood phosphorus

Nine publications (17, 19, 21, 34, 36, 37, 40, 42, 46) analyzed blood phosphorus levels in 411 patients (214 probiotics/synbiotics patients; 197 control patients). After comprehensive analysis, there was no statistically significant difference (SMD: −0.08; 95% CI: −0.28, 0.11; *p* = 0.41) or heterogeneity (I ^2^ = 0%, *p* = 0.96) in the change of blood phosphorus levels of the patients in the probiotics/synbiotics group compared with the control group (Figure 7C). Funnel plots (Figure 6G) and Egger’s test did not reveal significant publication bias (*p* = 0.503).

Based on an array of subgroup analyses, we did not observe an effect of probiotics/ synbiotics supplementation on blood phosphorus in hemodialysis individuals (k = 7, SMD: −0.05, 95% CI: −0.29, 0.2, I^2^ = 0%, *p* = 0.72) or non-hemodialysis individuals (k = 3, SMD: −0.14, 95% CI: −0.46, 0.18, I^2^ = 0%, *p* = 0.38). Based on geographical location, we observed no significant change of blood phosphorus in patients from countries located in Asia (k = 7; SMD: −0.08, 95% CI: −0.29, 0.14, I^2^ = 0%, p = 0.48). In terms of treatment time, we observed no significant change of blood phosphorus in both long term (k = 6; SMD: −0.16, 95% CI: −0.39, 0.08, I^2^ = 0%, *p* = 0.19) and short term (k = 4, SMD: 0.09, 95% CI: −0.26, 0.44, I^2^ = 0%, *p* = 0.62). In addition, no significant changes in blood phosphorus were observed with probiotics/synbiotics supplementation in individuals above and below 60 years of age (≥60 years, k = 2, SMD: −0.04, 95% CI: −0.57, 0.48, I^2^ = 7%, *p* = 0.87), (<60 years, k = 7, SMD: −0.03, 95% CI: −0.27, 0.22, I^2^ = 0%, *p* = 0.84; Table 2).

### 3.8 Sensitivity analysis

**Figure 8:**
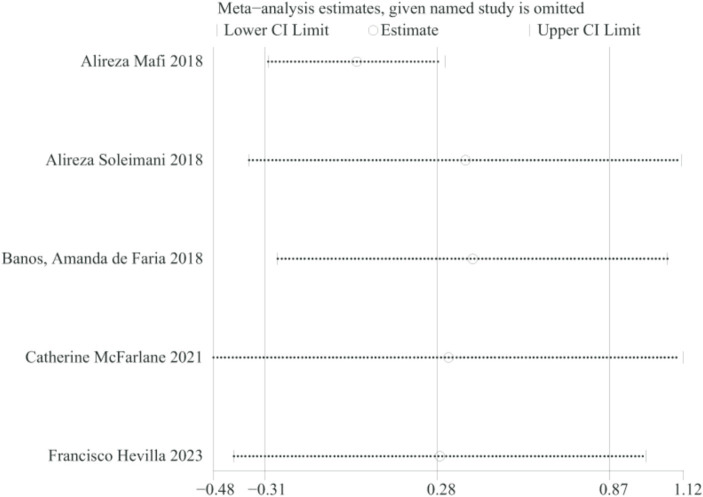
Sensitivity analysis of HDL.

Because the comprehensive analysis of HDL showed significant heterogeneity, we conducted one-way sensitivity analyses for comparison of HDL to evaluate the influence of each individual study on the combined SMD through removing the individual study one by one. Sensitivity analyses revealed that the new combined SMD remained constant after exclusion of any individual study for HDL (Figure 8).

## 4 Discussion

### 4.1 Findings from meta-analysis

The gut microbiota consists of more than 100 trillion bacteria and plays an important role in normal body functions, particularly in immune and metabolic homeostasis. There is growing evidence that alterations in the gut microbiota can affect multiple organ systems and lead to many chronic diseases such as CKD (48). CKD is a serious and steadily growing health problem worldwide. As a progressive disease, the majority of CKD patients are referred to dialysis treatment, and effective pharmacological treatments are still being explored (49). One of the promising drug candidates is the modification of dysbiotic gut flora through probiotics/synbiotics supplementation to reduce levels of gut-derived uremic toxins and reduce chronic microinflammation thereby improving renal function (50). Due to the small number of published studies, it is still highly controversial whether this probiotics/synbiotics intervention affects renal function, uremic toxicity, and inflammation levels in patients with CKD. In this study, we systematically compiled and analyzed the clinical evidence of RCT on probiotics/synbiotics for the treatment of CKD to provide better guidance for clinical practice.

Our results showed that probiotic/synbiotics supplementation of CKD patients can decreased BUN in CKD patients, and no significant heterogeneity was found in the analysis results, which to a certain extent reflects the effect of probiotic/synbiotics to improve renal function in CKD patients. However, the use of probiotic/synbiotics had no effect on eGFR and serum creatinine, indicators of renal function. And the analysis results of eGFR and serum creatinine did not show apparent heterogeneity. This result may be due to the fact that eGFR and serum creatinine is strongly influenced by ethnicity, gender and age. And the baseline could not be standardized across studies. In addition to this, the use of probiotics/synbiotics can reduce CRP expression levels and improve inflammatory status in CKD patients. However, probiotic/synbiotic supplementation did not significantly alter blood electrolyte levels and lipid metabolism-related markers in CKD patients compared with placebo. No significant publication bias was detected by Egger’s test and funnel plot for all indicators except blood calcium.

We also stratified patients according to region, duration of treatment, age, and whether or not they were receiving dialysis treatment. Subgroups were analyzed for several important indicators: urea nitrogen, serum creatinine, CRP, indoxyl sulfate, and blood phosphorus. The results of subgroup analyses suggested that probiotics/synbiotics were more effective in reducing BUN in non-hemodialysis CKD patients. This probably due to the fact that CKD in hemodialysis patients usually has progressed to the stage of end-stage renal disease, and the renal units are irreversibly damaged, which is difficult to be improved by drug treatment (51). The results of subgroup analysis also showed that probiotics/synbiotics had a better effect on CRP reduction in non-hemodialysis CKD patients, suggesting that probiotics/synbiotics had a better effect on the improvement of inflammation in non-hemodialysis patients. In CKD patients, increased inflammation is associated with several negative clinical outcomes such as increased oxidative stress, vascular dysfunction and increased risk of cardiovascular disease, as the ameliorative effect of probiotics/synbiotics on inflammation in non-hemodialysis patients is beneficial to patients (52, 53). And one of the goals of treatment for hemodialysis patients is to reduce inflammation, thus effectively improving the survival of these patients (54). Meanwhile, the results of the subgroup analysis stratified on the basis of the duration of probiotics/synbiotics treatment showed that there is a significant reduction in BUN and CRP levels in CKD patients when probiotics/synbiotics are applied for a longer period of time compared to the control group. This suggested that adherence to probiotics/synbiotics for a longer period of time is more favorable for CKD patients. Interestingly, when subgroup analyses were performed on a regional basis, we found that probiotics/synbiotics supplementation was more effective in reducing BUN in Asian patients and in reducing CRP in patients from the Europe. The number of studies that included patients from the Europe was small, so the related results need to be further verified.

### 4.2 Possible mechanisms

The relationship between probiotics/synbiotics and CKD has been recognized with the increasing understanding of the health effects of microbial balance on the host. Essentially, probiotics/synbiotics supplementation can modulate the imbalance of the gut microbiota for the biosynthesis of targeted compounds with bioactive properties in CKD patients (55). At the same time, probiotics improves the integrity of the intestinal epithelial barrier and reduces the production of uremic toxins to some extent (56, 57). With the fluctuation of gut bacteria, probiotics can modulate inflammation by establishing a balance between pro-inflammatory and anti-inflammatory cytokines in the body (58). In addition, metabolites from the gut microbiota also play an important role in maintaining homeostasis in the gut for the benefit of host health through fermentation of amino acids and dietary fiber, production of vitamins and neurotransmitters, and modification of bile acids (59). For example, Zhu et al. (60) showed that short-chain fatty acids (SCFAs) from a variety of bacteria reduced the expression of genes for inflammatory cytokines, chemokines, and pro-fibrotic proteins in diabetic kidneys, which, in turn, reduced proteinuria, glomerular hypertrophy, pedunculated cell injury, and interstitial fibrosis in mice with acute kidney injury and CKD. Indeed, probiotic or synbiotics supplementation may also reverse the expansion of harmful gut microbes that produce excess uremic toxins and attenuate the development of CKD (32, 61).

### 4.3 Strengths and limitations

#### 4.3.1 Limitations

Firstly, most of the publications included in this meta-analysis were RCT cohort studies. The sample sizes of RCT studies are small, and potential bias from small samples is unavoidable. Secondly, the main population groups of the studies we included were from Asia, with fewer people from other states, and there may be regional selectivity bias. Whether the results can be generalized to other regions needs to be confirmed by further studies. Thirdly, the heterogeneity of the studies included in the analysis of HDL was large, which may hinder the robustness of the results. In addition, the RCTs involved in the study did not report adverse events in patients, so adverse events were not included in the study.

#### 4.3.2 Strengths

Firstly, this study is the latest meta-analysis of probiotics/synbiotics for CKD with the largest sample size available. Secondly, the original studies included in this article were all RCTs, which were of high quality, with good study design and a balanced baseline. Thirdly, this study confirmed that probiotics/synbiotics had an ameliorative effect on renal function and inflammatory status in patients with CKD, which has been consistent with previous studies. Fourthly, compared with previous meta-analyses, our study included a wider range of outcome indicators and incorporated the most recent RCT studies, thus allowing for the most up-to-date evidence on probiotics/synbiotics supplementation in CKD treatment. What’s more, this study provides more options and guidance notes for clinical CKD treatment.

## 5 Conclusion

In conclusion, this is the latest systematic review and meta-analysis demonstrating that probiotic/synbiotics interventions reduced BUN and CRP in patients with CKD, although there was insufficient evidence of a positive effect of probiotics/synbiotics on lipids and blood electrolytes. Regarding BUN and CRP, the results of our meta-analysis emphasize the positive effects of probiotic/synbiotics supplementation using longer (≥3 months) treatment durations in Asian patients. This area deserves further research to elucidate the mechanism of probiotics/synbiotics for the possible treatment of CKD and to further assess the safety of different types of probiotics/synbiotics through randomized controlled trials.

## Data availability statement

The original contributions presented in the study are included in the article/Supplementary material, further inquiries can be directed to the corresponding author.

## Author contributions

CL: Formal analysis, Writing – original draft. LY: Writing – review & editing. WW: Investigation, Writing – review & editing. PF: Funding acquisition, Supervision, Writing – review & editing.
