# Supplementary material for: Efficacy of probiotics/synbiotics supplementation in patients with chronic kidney disease: a systematic review and meta-analysis of randomized controlled trials
Source: Front Nutr. 2024 Aug 6;11:1434613. doi: 10.3389/fnut.2024.1434613 (PMC11333927; doi:10.3389/fnut.2024.1434613)

Supplementary Material

**Table S1.**Search terms employed in the screening based on title, abstract and keywords in the literature search.

| **Database** | **Search terms** |  |
| --- | --- | --- |
|  |  |  |
| PubMed  Cochrane Library  Web of Science  Embase | ((("Probiotics"[Mesh]) OR (Probiotic)) OR (("Synbiotics"[Mesh]) OR (Synbiotic))) AND (("Renal Insufficiency, Chronic"[Mesh]) OR ((((((((((((((((((((Chronic Renal Insufficiencies) OR (Renal Insufficiencies, Chronic)) OR (Chronic Renal Insufficiency)) OR (Kidney Insufficiency, Chronic)) OR (Chronic Kidney Insufficiency)) OR (Chronic Kidney Insufficiencies)) OR (Kidney Insufficiencies, Chronic)) OR (Chronic Kidney Diseases)) OR (Chronic Kidney Disease)) OR (Disease, Chronic Kidney)) OR (Diseases, Chronic Kidney)) OR (Kidney Disease, Chronic)) OR (Kidney Diseases, Chronic)) OR (Chronic Renal Diseases))) OR (Chronic Renal Disease)) OR (Disease, Chronic Renal)) OR (Diseases, Chronic Renal)) OR (Renal Disease, Chronic)) OR (Renal Diseases, Chronic)))  ((Probiotics or Probiotic or (Synbiotics or Synbiotic)) and (Renal Insufficiency, Chronic or (Chronic Renal Insufficiencies or Renal Insufficiencies, Chronic or Chronic Renal Insufficiency or Kidney Insufficiency, Chronic or Chronic Kidney Insufficiency or Chronic Kidney Insufficiencies or Kidney Insufficiencies, Chronic or Chronic Kidney Diseases or Chronic Kidney Disease or Disease, Chronic Kidney or Diseases, Chronic Kidney or Kidney Disease, Chronic or Kidney Diseases, Chronic or Chronic Renal Diseases or Chronic Renal Disease or Disease, Chronic Renal or Diseases, Chronic Renal or Renal Disease, Chronic or Renal Diseases, Chronic)))  (((Probiotics) OR (Probiotic)) OR ((Synbiotics) OR (Synbiotic))) AND ((Renal Insufficiency, Chronic) OR ((((((((((((((((((((Chronic Renal Insufficiencies) OR (Renal Insufficiencies, Chronic)) OR (Chronic Renal Insufficiency)) OR (Kidney Insufficiency, Chronic)) OR (Chronic Kidney Insufficiency)) OR (Chronic Kidney Insufficiencies)) OR (Kidney Insufficiencies, Chronic)) OR (Chronic Kidney Diseases)) OR (Chronic Kidney Disease)) OR (Disease, Chronic Kidney)) OR (Diseases, Chronic Kidney)) OR (Kidney Disease, Chronic)) OR (Kidney Diseases, Chronic)) OR (Chronic Renal Diseases))) OR (Chronic Renal Disease)) OR (Disease, Chronic Renal)) OR (Diseases, Chronic Renal)) OR (Renal Disease, Chronic)) OR (Renal Diseases, Chronic))) (Topic) and Preprint Citation Index (Exclude – Database)  (((Probiotics) OR (Probiotic)) OR ((Synbiotics) OR (Synbiotic))) AND ((Renal Insufficiency, Chronic) OR ((((((((((((((((((((Chronic Renal Insufficiencies) OR (Renal Insufficiencies, Chronic)) OR (Chronic Renal Insufficiency)) OR (Kidney Insufficiency, Chronic)) OR (Chronic Kidney Insufficiency)) OR (Chronic Kidney Insufficiencies)) OR (Kidney Insufficiencies, Chronic)) OR (Chronic Kidney Diseases)) OR (Chronic Kidney Disease)) OR (Disease, Chronic Kidney)) OR (Diseases, Chronic Kidney)) OR (Kidney Disease, Chronic)) OR (Kidney Diseases, Chronic)) OR (Chronic Renal Diseases))) OR (Chronic Renal Disease)) OR (Disease, Chronic Renal)) OR (Diseases, Chronic Renal)) OR (Renal Disease, Chronic)) OR (Renal Diseases, Chronic))) |  |

**Figure S1.**Risk of bias.


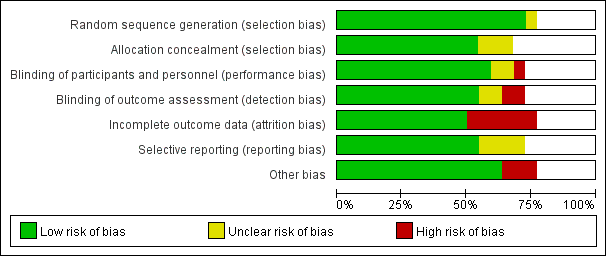


**Figure S2.**Summary of risk of bias.


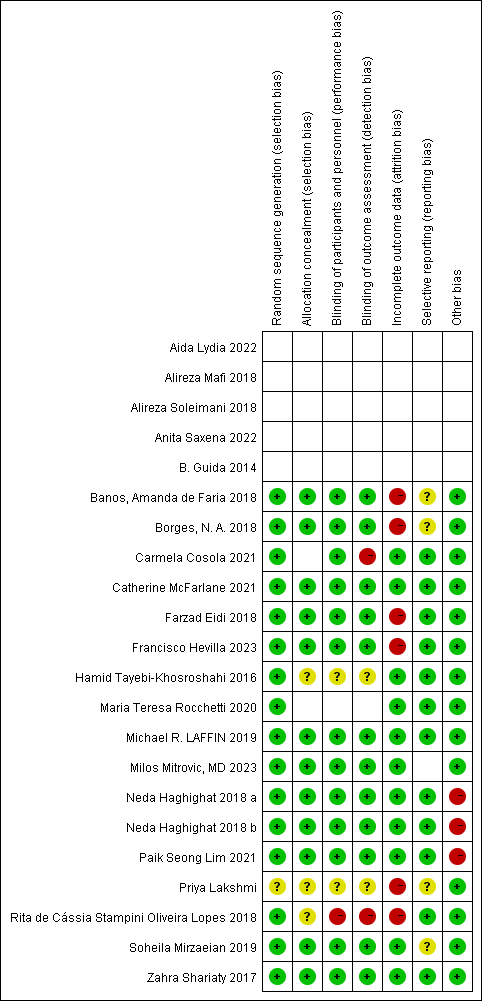

Supplement: Supplementary file 1 [file Data_Sheet_1.docx]
